# Nasal high-flow bronchodilator nebulization: a randomized cross-over study

**DOI:** 10.1186/s13613-018-0473-8

**Published:** 2018-12-20

**Authors:** François Reminiac, Laurent Vecellio, Laetitia Bodet-Contentin, Valérie Gissot, Deborah Le Pennec, Charlotte Salmon Gandonnière, Maria Cabrera, Pierre-François Dequin, Laurent Plantier, Stephan Ehrmann

**Affiliations:** 1 Médecine Intensive Réanimation, CHRU de Tours, 2, Bd Tonnellé, 37044 Tours Cedex 9, France; 2 Centre d’étude des pathologies respiratoires, INSERM U1100, Faculté de médecine, Université de Tours, Tours, France; 3 Clinique du Mail, La Rochelle, France; 4 Centre d’investigation clinique, INSERM CIC 1415, CHRU de Tours, Tours, France; 5 CRICS-TRIGGERSEP Network, Tours, France; 6 Service de pneumologie et d’explorations fonctionnelles respiratoires, CHRU de Tours, Tours, France

**Keywords:** Albuterol, Respiratory function tests, Nebulizers and vaporizers, Chronic obstructive pulmonary disease

## Abstract

**Background:**

There is an absence of controlled clinical data showing bronchodilation effectiveness after nebulization via nasal high-flow therapy circuits.

**Results:**

Twenty-five patients with reversible airflow obstruction received, in a randomized order: (1) 2.5 mg albuterol delivered via a jet nebulizer with a facial mask; (2) 2.5 mg albuterol delivered via a vibrating mesh nebulizer placed downstream of a nasal high-flow humidification chamber (30 L/min and 37 °C); and (3) nasal high-flow therapy without nebulization. All three conditions induced significant individual increases in forced expiratory volume in one second (FEV_1_) compared to baseline. The median change was similar after facial mask nebulization [+ 350 mL (+ 180; + 550); + 18% (+ 8; + 30)] and nasal high flow with nebulization [+ 330 mL (+ 140; + 390); + 16% (+ 5; + 24)], *p* = 0.11. However, it was significantly lower after nasal high-flow therapy without nebulization [+ 50 mL (− 10; + 220); + 3% (− 1; + 8)], *p* = 0.0009. FEV_1_ increases after facial mask and nasal high-flow nebulization as well as residual volume decreases were well correlated (*p* < 0.0001 and *p* = 0.01). Both techniques showed good agreement in terms of airflow obstruction reversibility (kappa 0.60).

**Conclusion:**

Albuterol vibrating mesh nebulization within a nasal high-flow circuit induces similar bronchodilation to standard facial mask jet nebulization. Beyond pharmacological bronchodilation, nasal high flow by itself may induce small but significant bronchodilation.

**Electronic supplementary material:**

The online version of this article (10.1186/s13613-018-0473-8) contains supplementary material, which is available to authorized users.

## Background

Nasal high-flow (NHF) therapy consists of delivering heated and humidified gas through a nasal cannula, at high flow rates, frequently exceeding patients’ inspiratory flow. This non-invasive respiratory support is increasingly used, particularly among hypoxemic critically ill patients as those high oxygen flow rates very efficiently improve oxygenation and reduce the rate of intubation [1, 2]. Nebulization is a technique used to deliver inhaled drugs directly acting on the respiratory tract. In critically ill patients, nebulization is very frequently used, in particular among patients undergoing non-invasive respiratory support [3]. The most frequently delivered inhaled drugs are bronchodilators, such as albuterol, provided to approximately 20% of patients in intensive care [3]. Thus, one may question the best way to combine the two therapies in order to deliver inhaled bronchodilators to patients undergoing NHF therapy. Indeed, NHF may be especially beneficial to patients suffering obstructive pulmonary disease for whom inhaled bronchodilator delivery is a cornerstone of therapy [4–6]. NHF washes out the anatomical dead space clearing exhaled carbon dioxide, and this may have benefit to patients with hypercapnia [7, 8]; it induces a positive end-expiratory pressure, a reduction in respiratory rate and increase in tidal volume, which all potentially lead to a reduction in the work of breathing among patients with dynamic hyperinflation [9, 10]; it enables precise control of the inspired fraction of oxygen to avoid excessive delivery among patients with chronic hypercapnia and altered respiratory drive; it ensures high humidification of inhaled gases favouring mucus hydration and thus clearance and is a very well-tolerated oxygen delivery method. Nevertheless, NHF merely represents an obstacle impeding inhaled drug delivery. Indeed, high gas flow rate and associated turbulent flow, high gas humidity, geometric angulation of the nasal cannula, and the nose anatomy physiologically retaining inhaled particles all represent hurdles to efficient inhaled drug delivery through an NHF circuit. In vitro data showed that when placing a vibrating mesh nebulizer close to the humidification chamber and limiting the system flow rate at 30 L/min, significant amounts of drug may be delivered to the respiratory tract [11–15]. That data have been confirmed by in vivo evaluation in a paediatric animal model and in adult radiolabelled deposition studies [16, 17]. Although uncontrolled case series are in favour of a clinically significant bronchodilation after delivery of albuterol through an NHF circuit, no controlled data are available in humans [18].

The objective of this study was to investigate the effect of vibrating mesh nebulized albuterol delivered through an NHF circuit on respiratory system mechanics as compared to Standard-nebulization using a jet nebulizer with a facial mask and NHF delivered without inhaled albuterol in a randomized controlled fashion.

## Methods

The study was approved by the institutional review board (Comité de Protection des Personnes Ouest-1, 2016-R6-PHAO15-SE/Airvoneb-2016-A00064-47, NCT02812979). Adult patients with reversible obstructive lung disease defined as a baseline of forced expiratory volume in one second (FEV_1_) over vital capacity ratio below 70% and a positive bronchodilator reversibility test (FEV_1_ increase of at least 12% and 200 mL after inhaled albuterol delivery [19]) as assessed in the past month were included after written informed consent. Non-inclusion criteria were ongoing exacerbation, hemoptysis, uncontrolled asthma, recent pneumothorax, lung or pleural biopsy, broncho-alveolar lavage, pregnancy, breast feeding, trusteeship, guardianship and albuterol allergy or intolerance. Patients underwent, on three separate days within 1 week, in a randomized order: (1) albuterol nebulization through a facial aerosol mask (Standard-nebulization), (2) albuterol nebulization within an NHF circuit (NHF-nebulization) and (3) sham nebulization within an NHF circuit (Control-NHF). Patients were instructed not to smoke or to take short- or long-acting bronchodilators, respectively 4, 6 and 12 h prior to each procedure.

### Standard-nebulization

2.5 mg albuterol (albuterol sulphate 2.5 mg/2.5 mL, Mylan N.V., Canonsburg, PA, USA) was placed in a jet nebulizer connected to a bucco-nasal facial mask positioned on the patient and driven with 6 L/min of non-heated and non-humidified pressurized air (Cirrus2 nebulizer and Adult EcoLite™ Aerosol Mask, both from Intersurgical, Wokingham, UK).

### NHF-nebulization

**Fig. 1:**
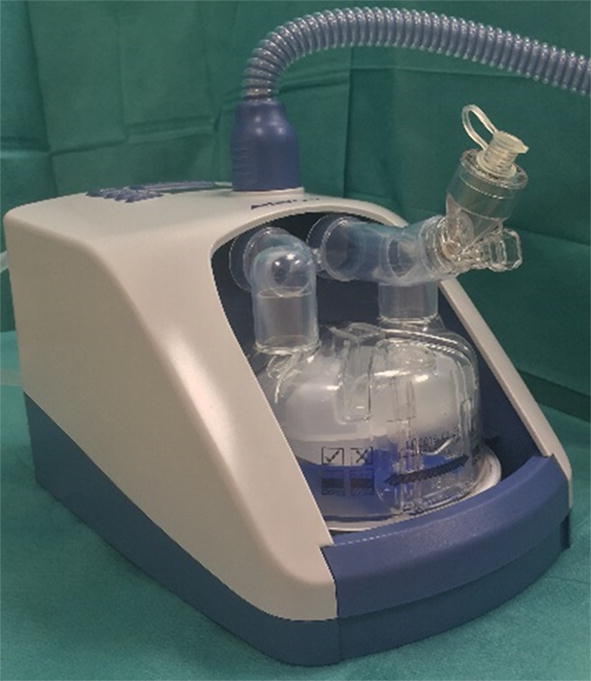
Nasal high flow nebulization set-up

2.5 mg albuterol was placed in a vibrating mesh nebulizer (Aerogen Solo^®^, Aerogen Ltd., Galway, Ireland) positioned immediately downstream of the humidification chamber of an NHF system (Airvo™2, Fisher & Paykel Healthcare, Auckland, New Zealand), using the Airvo™Neb connector (Fig. 1). NHF was set at 30 L/min of air with 100% relative humidity at 37 °C using medium size nasal cannula. The NHF session lasted 30 min, and nebulization was started after 10 min of NHF therapy.

### Control-NHF

The patient was placed under NHF during 30 min in the same conditions as for NHF-nebulization with an empty nebulizer. The patient was kept blind between the NHF-nebulization and Control-NHF procedures.

The primary outcome was the relative increase in FEV_1_ after NHF-nebulization as compared to Standard-nebulization. Pulmonary function tests (spirometry and plethysmography; calibrated Jaeger MasterScreen body plethysmograph, Spirometry SentrySuite v2.10, CareFusion, Rolle, Switzerland) were performed before and after each procedure, according to guidelines [19]. Spirometry was started immediately after the end of the 30 min NHF sessions, at least 10 min after the end of nebulization. Pulmonary function tests were performed following the same time span after the end of NHF therapy in both conditions comprising NHF (NHF-nebulization and Control-NHF) and following the same time span after the end of nebulization in both conditions comprising active nebulization (Standard-nebulization and NHF-nebulization).

Plethysmography loops were evaluated, and patients were classified as presenting expiratory flow limitation or not [20, 21]. Volumetric capnography was performed before and after each procedure (5 duplicate measurements, patients breathing out at slow and steady flow from maximal inspiration to maximal expiration) and the slope of the third phase of the capnogram measured. All pulmonary function tests were performed and interpreted by investigators and technicians blind to the procedure randomization. Patients’ comfort was recorded using a visual analogical scale (range 0–100, with higher scores indicating higher comfort). The NHF-nebulization set-up and vibrating mesh nebulizer performance were tested in vitro prior to the clinical study (see Additional file 1).

### Statistical analysis

The sample size calculation was based on previous data which showed a standard deviation of FEV_1_ of 10% of the baseline value [22]. Taking into account the cross-over design, this non-inferiority trial, testing the hypothesis that NHF-nebulization is non-inferior to Control-nebulization in terms of FEV_1_ relative increase, with a non-inferiority margin of 8%, a unilateral alpha risk of 2.5% and a beta risk of 10%, had to enrol 24 patients.

An association between the randomization order and primary outcome was assessed looking for interaction between the relative increase in FEV_1_ and the procedure position to rule out a carry-over or learning effect on pulmonary function tests (nonparametric Kruskal–Wallis test). To partition the increase in FEV_1_ potentially due to NHF alone from the pharmacological effect of albuterol nebulization, the FEV_1_ increase attributable to albuterol nebulization was calculated individually, by subtracting Control-NHF-induced absolute FEV_1_ increase from NHF-nebulization-induced absolute FEV_1_ increase.

Quantitative variables were expressed as median and interquartile range and were compared before and after each procedure using a Wilcoxon signed rank test. Individual changes (before/after the procedure) were compared between procedures (Standard-nebulization, NHF-nebulization and Control-NHF) using the nonparametric Friedman test accounting for the cross-over design, and if significant, two-by-two comparisons were performed with the Wilcoxon signed rank test. Correlation between quantitative variables was evaluated with the Spearman correlation coefficient. Qualitative variables were expressed as counts and percentages. The agreement between Standard-nebulization and NHF-nebulization in terms of airway obstruction reversibility (200 mL absolute and 12% relative increase in FEV_1_ [19]) was assessed using the kappa coefficient. A *p* value < 0.05 was considered significant.

## Results

In vitro results are presented in the Additional file 1. From June 2016 to April 2018, 11,288 patients underwent pulmonary function tests, 4905 spirometry with plethysmography, and beta-2-adrenergic agonist-induced reversibility was tested in 3552 patients of which 25 were included (Table 1).

**Table 1:** Patients’ baseline characteristics

Variable	*N* = 25
Female/male	10 (40%)/15 (60%)
Age (years)	60 (53; 68)
Main respiratory disease	
Asthma	9 (36%)
COPD	14 (56%)
Other	2 (8%)
Height (cm)	169 (165; 176)
Weight (kg)	75 (64; 80)
Body mass index (high/weight^2^)	26 (23; 29)
FEV_1_ (L)	1.83 (1,38; 2,03)
Percentage of predicted (%)	60 (53; 71)
FEV_1_/vital capacity (%)	54 (45; 60)
Functional residual capacity (L)	5,0 (3,9; 6,0)
Percentage of predicted (%)	150 (139; 171)
Residual volume (L)	4,0 (2,9; 4,4)
Percentage of predicted (%)	172 (154; 184)
Presence of expiratory flow limitation	6 (24%)

Data are presented as count (percentage) and median (interquartile range)

*COPD* chronic obstructive pulmonary disease, *FEV*_*1*_ forced expiratory volume in one second

### FEV_1_ change

**Fig. 2:**
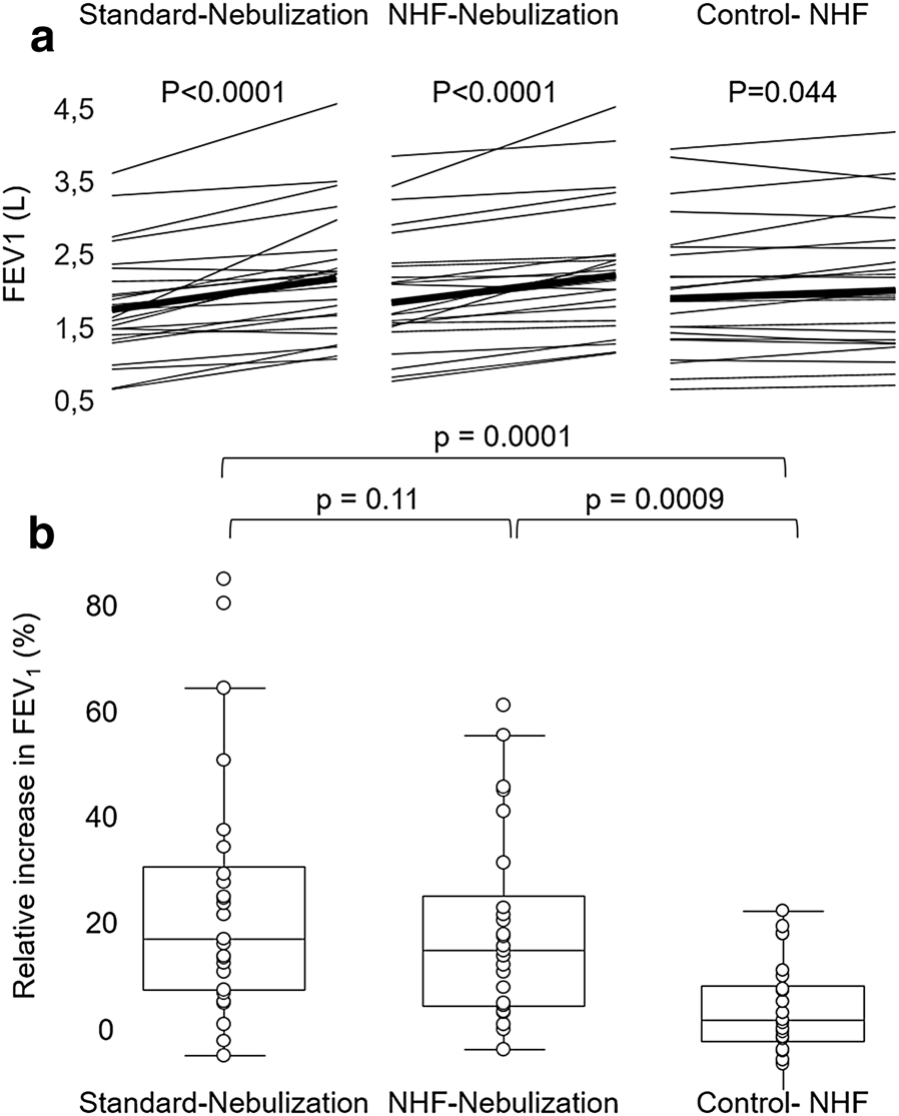
Individual change in forced expiratory volume in one second. **a** Individual values of forced expiratory volume in one second are indicate before and after each procedure at the left and right of each panel, respectively. The thick line represents the median values of the population. **b** Relative changes in forced expiratory volume in one second were similar and not significantly different between Standard-nebulization and nasal high-flow nebulization, whereas changes were significantly lower when implementing nasal high-flow without nebulization. Standard-nebulization consisted in 2.5 mg albuterol delivery with a jet nebulizer connected to an aerosol facial mask, nasal high-flow nebulization consisted in 2.5 mg albuterol delivered within a nasal high-flow circuit, and Control-nasal high-flow consisted in nasal high-flow delivered without nebulization. *NHF* nasal high-flow, *FEV*_*1*_ forced expiratory volume in one second

After Standard-nebulization, FEV_1_ significantly increased from 1.77 L (1.43; 2.16) to 2.20 L (1.69; 2.47), *p* < 0.0001 (Table 2). Individual absolute and relative increases in FEV_1_ were, respectively, 350 mL (180; 550) and 18% (8; 30). NHF-nebulization similarly induced a significant FEV_1_ increase: 1.77 L (1.47; 2.27) to 2.14 L (1.71; 2.41), *p* < 0.0001, with individual absolute and relative increases of 330 mL (140; 390) and 16% (5; 24): Fig. 2.

**Table 2:** Spirometry, plethysmography and volumetric capnography results

	Standard-nebulisation			NHF-nebulization			Control-NHF		
	Before	After	Individual change	Before	After	Individual change	Before	After	Individual change
FEV_1_ (L)	1.77 (1.43; 2.16)	2.20 (1.69; 2.47)	0.350 (0.180; 0.550)* 18% (8; 30)*	1.77 (1.47; 2.27)	2.14 (1.71; 2.41)	0.330 (0.140; 0.390)* 16% (5; 24)*	1.83 (1.36; 2.42)	1.93 (1.27; 2.52)	0.050 (− 0.010; 0.220)* 3% (− 1; 8)*
Functional residual capacity (L)	4.58 (3.89; 5.22)	4.07 (3.42; 4.88)	− 0.33 (− 0.71; − 0.17)*	4.42 (3.67; 5.35)	4.04 (3.45; 5.09)	− 0.40 (− 0.64; − 0.12)*	4.58 (3.80; 5.38)	4.42 (3.72; 5.53)	− 0.02 (− 0.24; 0.10)
Residual volume (L)	3.42 (2.63; 4.22)	2.89 (2.42; 3.54)	− 0.37 (− 0.82; − 0.12)*	3.22 (2.53; 4.29)	2.90 (2.52; 4.20)	− 0.34 (− 0.64; − 0.06)*	3.27 (2.76; 3.99)	3.19 (2.72; 4.56)	− 0.09 (− 0.34; 0.16)
Forced vital capacity (L)	3.57 (2.66; 4.39)	3.65 (3.15; 4.59)	0.32 (0.08; 0.57)*	3.41 (2.79; 4.37)	3.51 (3.05; 4.47)	0.11 (0.00; 0.34)*	3.28 (2.74; 4.52)	3.58 (2.64; 4.42)	0.10 (− 0.10; 0.25)
Plethysmographic airway resistances (raw)	5.31 (3.72; 6.94)	2.89 (2.54; 3.80)	− 2.06 (− 3.82; − 0.96)*	4.62 (3.48; 7.27)	3.10 (2.39; 3.79)	− 1.89 (− 3.36; − 0.69)*	4.71 (3.22; 7.02)	4.64 (2.86; 5.77)	− 0.39 (− 1.02; 0.04)*
Inspiratory capacity (L)	2.36 (2.03; 2.71)	2.63 (2.26; 3.34)	0.30 (0.14; 0.54)*	2.59 (2.12; 2.90)	2.72 (2.17; 3.17)	0.20 (0.05; 0.47)*	2.21 (1.78; 2.97)	2.61 (1.93; 2.91)	0.10 (− 0.07; 0.20)
Part III of the volumetric capnography slope (*n* = 16)	0.56 (0.47; 0.74)	0.66 (0.51; 0.92)	0.04 (− 0.03; 0.13)	0.67 (0.41; 0.94)	0.64 (0.40; 0.98)	0.03 (− 0.11; 0.08)	0.62 (0.47; 0.89)	0.65 (0.45; 0.91)	0.01 (− 0.05; 0.12)

Standard-nebulization consisted in 2.5 mg albuterol delivery with a jet nebulizer connected to an aerosol facial mask; NHF-nebulization: 2.5 mg albuterol delivered within a nasal high-flow (NHF) circuit; Control-NHF: nasal high flow without nebulization

*FEV*_*1*_ forced expiratory volume in one second, *NHF* nasal high-flow

*p < 0.05 for individual changes before and after each session with one technique

After Control-NHF without bronchodilator delivery, FEV_1_ increased from 1.83 L (1.36; 2.42) to 1.93 L (1.27; 2.52), *p* = 0.044: Fig. 2. Median individual absolute and relative increases were 50 mL (− 10; 220) and 3% (− 1; 8): Table 2.

**Fig. 3:**
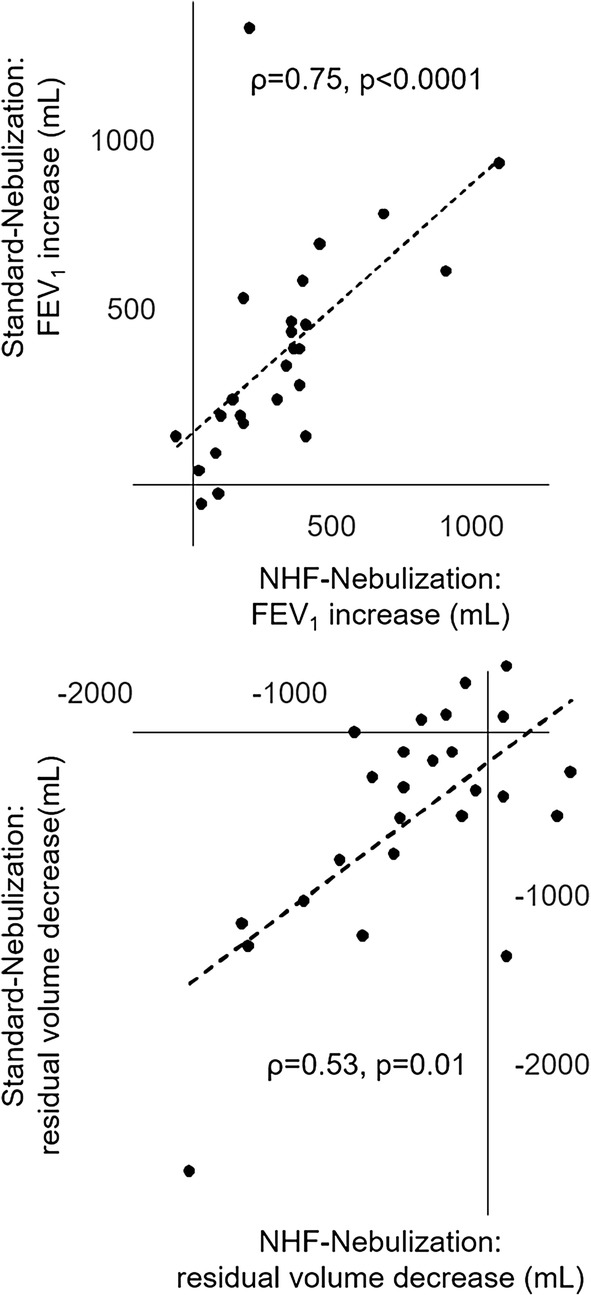
Correlation of lung mechanics changes induced by Standard-nebulization and Nasal high-flow nebulization. Changes in forced expiratory volume in one second (FEV_1_) and in residual volume after 2.5 mg albuterol nebulization with a standard facial mask jet nebulizer and with a vibrating mesh nebulizer place within a nasal high-flow circuit were well correlated. *NHF* nasal high-flow, *FEV*_*1*_ forced expiratory volume in one second

No interaction was observed between the randomization order of the procedures and the absolute and relative increase in FEV_1_ (*p* = 0.66 and *p* = 0.59, respectively). There was an overall statistically significant difference between procedures for the absolute and relative increase in FEV_1_ (*p* < 0.001 and *p* = 0.001, respectively). In two-by-two comparisons, changes in FEV_1_ after NHF-nebulization and Standard-nebulization were not significantly different (Fig. 2) and well correlated (Fig. 3) and exhibited low bias (Figure E3 of the Additional file 1). Changes in FEV_1_ after Control-NHF were significantly lower (Fig. 2). Of note, when calculating changes attributable to albuterol nebulization during NHF-nebulization (subtracting Control-NHF-induced changes from NHF-nebulization-induced changes), the individual absolute increase in FEV_1_ attributable to albuterol nebulization was 230 mL (− 45; 385), a value significantly lower than the FEV_1_ increase after Standard-nebulization (*p* = 0.009).

### Airflow obstruction reversibility

Of the 18 patients with an increase in FEV_1_ of more than 200 mL after Standard-nebulization during study measurements, 14/18 (78%) also showed such an increase after NHF-nebulization. Seventeen patients had an increase in FEV_1_ of more than 12% after Standard-nebulization, of these 15/17 (88%) did so after NHF-nebulization. Combining both criteria according to guidelines (absolute and relative increase in FEV_1_ [19]), 16 patients met the criteria for airway obstruction reversibility after albuterol Standard-nebulization during study measurements, all but two of these (*n* = 14/16, 88%) met the criteria after albuterol NHF-nebulization; conversely, all but one of the patients meeting the criteria after NHF-nebulization (13/14 93%) did so after Standard-nebulization (kappa 0.60, 95% confidence interval 0.29–0.90).

Of note, after Control-NHF, 8/25 patients (32%) had an FEV_1_ increase of at least 200 mL and 5/25 (20%) of at least 12%. Five patients (20%) met the criteria for airway obstruction reversibility after Control-NHF without the addition of a bronchodilator drug [19]. See Additional file 1: Table E2 for details on those patients. No association was observed between expiratory flow limitation observed on plethysmographic loop inspection (observed in 6 patients) and positive response in terms of FEV_1_ increases after Control-NHF, as only one flow limited patient showed such a positive response.

### Other pulmonary function tests

Plethysmography showed significant improvement in lung volumes after Standard-nebulization and NHF-nebulization (Table 2). Significant individual reduction in functional residual capacity was observed after NHF-nebulization: from 4.42 L (3.67; 5.35) to 4.04 L (3.45; 5.09)—individual changes − 400 mL (− 640; − 120), *p* = 0.001. This change was correlated with changes in residual volume observed after Standard-nebulization: Fig. 3. When NHF was delivered without albuterol nebulization, no such significant volume changes occurred (Table 2). Significant changes in plethysmography-measured airway resistances also occurred after Standard-nebulization, NHF-nebulization and Control-NHF (Table 2). The third part of the expired volumetric capnogram, which could be measured for all procedures in sixteen patients, did not show a significant change after either procedure, and individual changes were not significantly different between procedures (*p* > 0.05).

### Tolerance

Overall tolerance of the NHF therapy and nebulization was excellent. No side effects were recorded during NHF-nebulization; one patient complained of moderate reversible dyspnoea during Standard-nebulization and during Control-NHF. No clinically significant changes in heart rate and respiratory rate occurred; individual changes were not statistically different between procedures (data not shown). Comfort, as measured by the visual analogical scale, was not significantly different between procedures, 85 (77; 96), 85 (65; 93) and 82 (66; 92) for Standard-nebulization, NHF-nebulization and Control-NHF, respectively (*p* = 0.34).

## Discussion

In patients with reversible obstructive pulmonary disease, away from an exacerbation, albuterol delivered by vibrating mesh nebulization through an NHF circuit appeared non-inferior to standard facial mask jet nebulization in terms of FEV_1_ increase. This was in part due to a small but a significant increase in FEV_1_ due to NHF without the addition of bronchodilator nebulization. To the best of our knowledge, this is the first controlled study in adults documenting clinical efficacy of nebulization within an NHF circuit adequately controlling for all confounding factors. These results have important clinical implications. As the use of NHF is expanding, physicians will increasingly be faced with patients undergoing NHF and requiring inhaled bronchodilator therapy [23]. Given the lack of controlled data, interrupting NHF therapy to deliver the inhaled medication may currently be the preferred option; these results show that albuterol can be delivered within the NHF circuit with the same efficacy and tolerance avoiding cumbersome equipment switches. These results are in line with the study of Bräunlich et al. who used a homecare NHF device to deliver a combination of albuterol and ipratropium bromide placing a jet nebulizer close to the nasal cannula but lacked a control group without nebulization [24]. Of note, positioning the nebulizer close to the nasal cannula may be suboptimal, as it favours aerosol deposition in the cannula. This deposition reduces drug delivery to the patient but was also associated with aerosol nasal dripping which may impact patients’ comfort [11]. Our results provide controlled evidence supporting the observation made by Morgan et al. of efficient albuterol delivery after nebulization within a NHF circuit set-up similar to the present one among children with acute bronchiolitis [18].

Effects of NHF without bronchodilator nebulization on pulmonary function tests are of complex interpretation. We observed a statistically significant increase in FEV_1_ after Control-NHF, albeit modest in magnitude (median increase of 50 mL and 3%, values below validated thresholds to define reversibility [19]); this result supports the hypothesis of an NHF-induced bronchodilation. Interestingly, 20% of the patients showed significant increases in FEV_1_ after Control-NHF meeting guideline criteria for airflow obstruction reversibility without having received a bronchodilator. Of note, FEV_1_ was measured after interruption of NHF in patients breathing spontaneously unlike other physiological studies which observed an increase in lung volumes measured during NHF therapy [25]. This may also explain the lack of association between flow limitation and FEV_1_ increase after Control-NHF. Plethysmography-measured lung volumes were not significantly affected by NHF in the present study. One can speculate on potential mechanism such as positive airway pressure and improved mucus hydration during the 30-min NHF session leading to the significant increase in FEV_1_ once the therapy is interrupted. Indeed, improved mucus clearance may lead to improved lung mechanics; however, no major cough and expectoration was observed among the included patients. NHF may also induce changes in respiratory pattern potentially leading to higher tidal volume and eventually to deeper inspiration during spirometry manoeuvres. Such mechanisms will need to be investigated in the future, particularly given the ongoing studies evaluating NHF among patients suffering obstructive pulmonary disease. This study has important limitations. Only stable patients were included; thus, extrapolation to the acute care setting of unstable decompensated patients warrants evaluation. Results cannot be extrapolated to other pharmacological classes, as the favourable results observe here in terms of nebulization efficiency during NHF are due in part to the large therapeutic index of albuterol [26]. Deposition studies performed in humans suggest other drugs like antibiotics are unlike effective when inhaled through an NHF circuit [17]. Clinical efficacy studies are required in intensive care unit, emergency department and pulmonology ward patients. Two different nebulizers were used in the study. We aimed to compare usual practice (facial mask jet nebulization) to the new modality of NHF-nebulization using a vibrating mesh nebulizer. Jet nebulization within the NHF circuit, albeit feasible, comes with important limitations as the gas driving the nebulizer interferes with the NHF oxygen content, humidity and temperature. Vibrating mesh facial mask nebulization is currently of uncommon practice. Thus, the potential limit of using different nebulizers represents a pragmatic choice favouring clinical applicability of the results. Using jet nebulization in combination with nasal high-flow therapy would need further evaluation. Of note, the study did not comprise a condition of sham jet nebulization to delineate individual effect of beta-2-adrenergic agonist nebulization per se. Only one NHF setting (temperature, flow rate, cannula size) and one dose of albuterol were evaluated clinically. However, results in other conditions, tested in the bench study, can give indications of potential dose adjustments, in case of nebulization with higher flow rates for example. The significantly improved delivery observed in vitro with non-humidified settings allows for new innovation in NHF devices to improve combined inhaled drug delivery.

In conclusion, the present work shows that albuterol vibrating mesh nebulization within an NHF circuit induces similar FEV_1_ increases and patient comfort and tolerance compared to standard facial mask jet nebulization and can be implemented in clinical practice. Beyond pharmacologically induced bronchodilation, NHF by itself may induce a small but significant increase in FEV_1_ which deserves further evaluation.

## Additional file

**Additional file 1.** In vitro testing and additional clinical results.

## Abbreviations

NHF: nasal high-flow
FEV_1_: forced expiratory volume in one second
Standard-nebulization: albuterol nebulization through a facial aerosol mask
NHF-nebulization: albuterol nebulization within a nasal high-flow circuit
Control-NHF: sham nebulization within a nasal high-flow circuit
