# Supplementary material for: Nasal high-flow bronchodilator nebulization: a randomized cross-over study
Source: Ann Intensive Care. 2018 Dec 20;8:128. doi: 10.1186/s13613-018-0473-8 (PMC6301902; doi:10.1186/s13613-018-0473-8)
Supplement: Supplementary file 1 — Additional file 1. In vitro testing and additional clinical results. [file 13613_2018_473_MOESM1_ESM.docx]

**Nasal high flow bronchodilator nebulization: a randomized cross-over study**

François REMINIAC, MD, PhD; Laurent VECELLIO, PhD; Laetitia BODET-CONTENTIN, MD, PhD; Valérie GISSOT, MD; Deborah LE PENNEC, MSc; Charlotte SALMON GANDONNIERE, MD; Maria CABRERA, MSc; Pierre-François DEQUIN, MD, PhD; Laurent PLANTIER, MD, PhD; Stephan EHRMANN, MD, PhD

**Additional file**

***In vitro* evaluation of the Nasal High-Flow (NHF) nebulization setup:**

The aim of this preliminary bench study was to validate the NHF-nebulization setup as previous bench studies evaluated NHF systems different from the Airvo2® (Fisher & Paykel, Auckland, New Zealand), device. In particular the specific connection piece enabling to connect a vibrating mesh nebulizer to the system places the nebulizer immediately downstream of the humidification chamber, a position slightly different from most available bench studies on the subject. Further mechanistic exploratory measurements were also performed varying the humidification, temperature and flow rate settings of the system.

**Methods and Materials:**

Experimental conditions: the Airvo2® (Fisher and Paykel Healthcare, Auckland, New Zealand) was used with an adult breathing circuit (900PT551) and medium size nasal cannula (OPT844).

Nebulization efficiency was assessed measuring drug output at the cannula outlet in different conditions:

1- Dry and ambient temperature settings: the heating plate of the Airvo2® was switch off and the humidification chamber was not filled with water.

2- Dry but warm setting: the heating plate of the Airvo™2 was switch on (34°C and 37°C were tested) but the humidification chamber was not filled with water.

3- Wet and warm setting (normal operating condition, used in the clinical study): the heating plate of the Airvo2® was switch on (34°C and 37°C were tested) and the humidification chamber filled with water.

For each setting, 4 flow rates were assessed in a randomized order: 15 L/min, 30 L/min, 45 L/min and 60 L/min.

At the beginning of each experiment and before nebulization, the temperature and relative humidity were recorded at the outlet of the circuit and in the room.

Six experiments were performed for each condition.

Nebulization procedure: a vibrating mesh nebulizer (Aerogen Solo®, Aerogen, Galway, Ireland) was connected to the outlet of the humidification chamber with a specific 3D printed prototype connector provided by Fisher and Paykel.

5 nebulizers were used for the whole set of *in vitro* measurements. To avoid any intra- or inter-batch inconsistencies, all 30 nebulizers used for the *in vitro* and clinical study underwent laser diffraction granulometry measurement (Spraytech, Malvern Instruments, Malvern, United Kingdom) testing 1 mL of 0.9% NaCl.

Experiments were performed loading 2.5 mL of albuterol sulfate (5mg/2.5mL, batch: Y2106, exp.: 03/2017, Mylan N.V., Canonsburg, PA, United States) in the nebulizers. The time necessary for complete nebulization was recorded.

Aerosol recovery at the cannula outlet: the nasal cannula was connected to artificial nares, allowing aerosol emitted from the cannula to be collected on a filter (PARI Gmbh, Starnberg, Germany).

**Figure E1:** Artificial nares and filter to collect aerosol delivered at the cannula outlet:


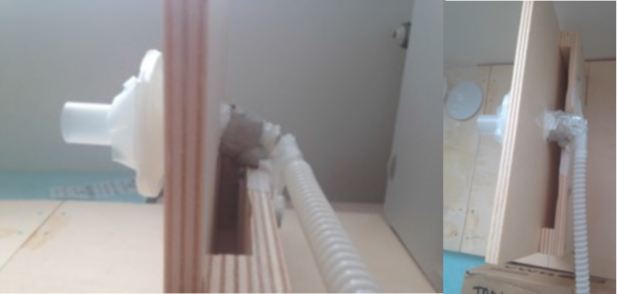


The mass of albuterol collected on the filter was eluted with 0.1M sodium hydroxide (NaOH) and then measured by spectrophotometry.

Analysis: the influence of nebulization settings and of the Airvo2® flow rates with a two-way analysis of variance followed, if significant by post hoc pairwise comparisons. Results were expressed as a percentage of the nebulizer charge (mean±standard deviation). A p value below 0.05 was considered significant. No adjustments for multiple comparisons were made.

**Results:**

All tested nebulizers showed consistent performance: mass median aerodynamic diameter of 4.62±0.52 µm, percentage of particles less than 5 µm in diameter 55±7%, nebulizer output 0.40±0.07 mL/min. Amounts of drug delivered at the cannula outlet in the different experimental conditions are presented in Table E1.

| **Table E1:** Aerosol masses delivered at the cannula outlet | | | | | |
| --- | --- | --- | --- | --- | --- |
|  | Non-humidified | | | Active humidification | |
| Temperature | 22°C | 34°C | 37°C | 34°C | 37°C  Clinical study conditions |
| NHF flow rate |  |  |  |  |  |
| 15 L/min | 19.6±2.2% | 31.4±2.2% | 37.2±3.9% | 23.3±3.4% | 25.4±4.8% |
| 30 L/min | 25.5±2.7% | 40.2±2.8% | 40.9±4.7% | 26.9±1.5% | 27.0±2.2% |
| 45 L/min | 26.0±4.2% | 34.4±2.5% | 39.0±2.6% | 21.2±3.3% | 23.7±2.8% |
| 60 L/min | 24.34±2.6% | 30.8±2.3% | 33.7±2.0% | 17.5±0.90% | 18.6±1.5% |
| NHF: nasal high flow. Values are indicated as mean±SD of six independent experiments and expressed as a percentage of the mass of drug charged in the nebulizer. Non-humidified condition refers to the humidification chamber being not filled with water and the NHF system run at the indicated temperature. Active humidification denotes normal functioning of the NHF system. | | | | | |

The conditions implemented in the clinical study (37°C active humidification at 30 L/min) were associated with 27.0±2.2% of the nebulizer charge being delivered at the cannula outlet. This amount of drug delivered was higher than in experimental conditions with flow rates higher or lower than 30 L/min, not significantly different from amounts delivered at 34°C active humidification and lower than amounts delivered in non-humidified conditions: Figure E2.

**Figure E2:** Drug delivered at the cannula outlet in 34°C and 37°C active humidification conditions


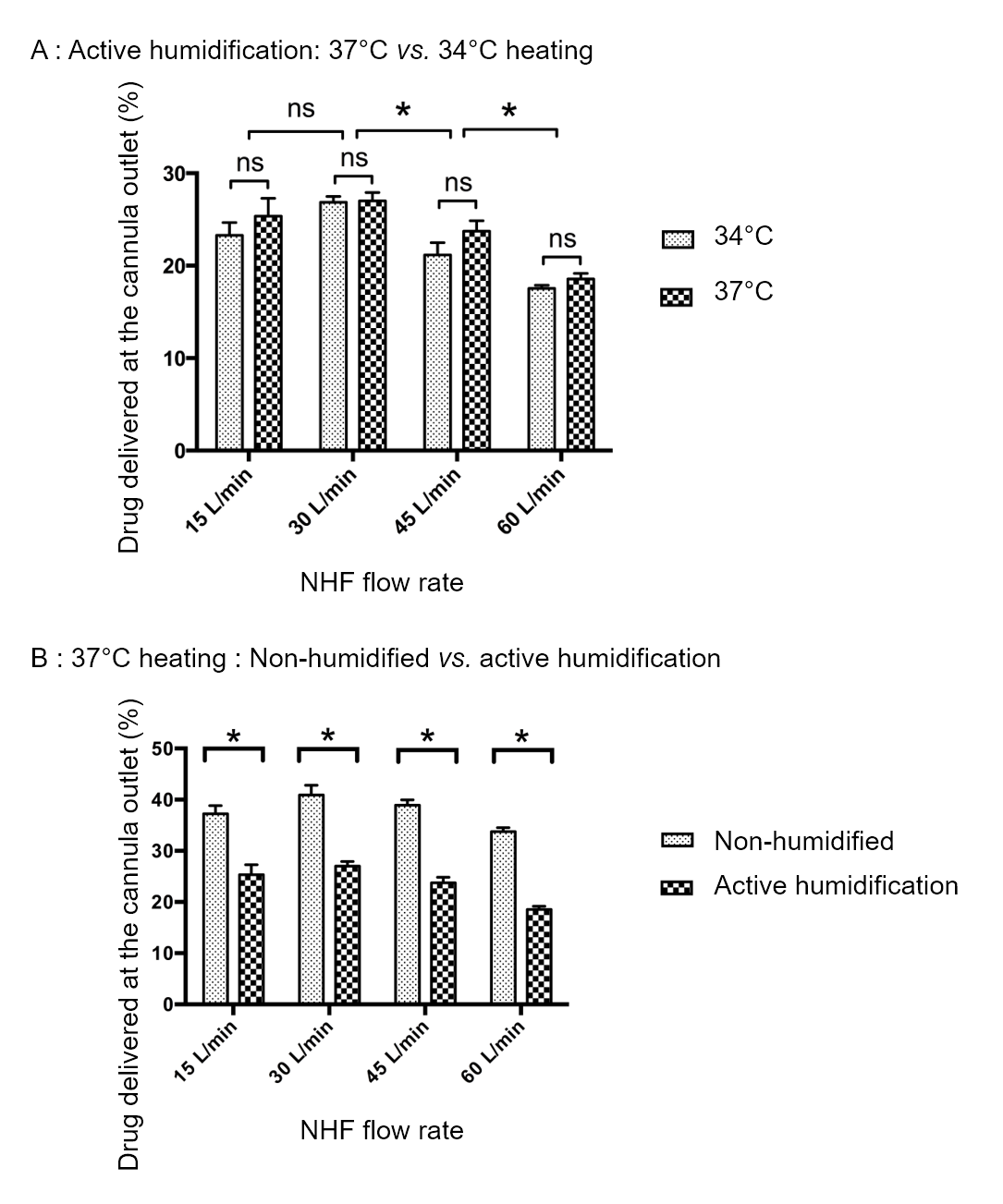


NHF: nasal high flow. Values are indicated as mean, error bars indicating standard deviation, of six independent experiments. Amount of drug delivered was expressed as a percentage of the nebulizer charge. Non-humidified condition refers to the humidification chamber being not filled with water and the NHF system run at the indicated temperature. Active humidification denotes normal functioning of the NHF system.

Figure E3: Bland & Altman representation of individual changes in lung mechanics after Nasal High Flow-Nebulization and Standard-Nebulization


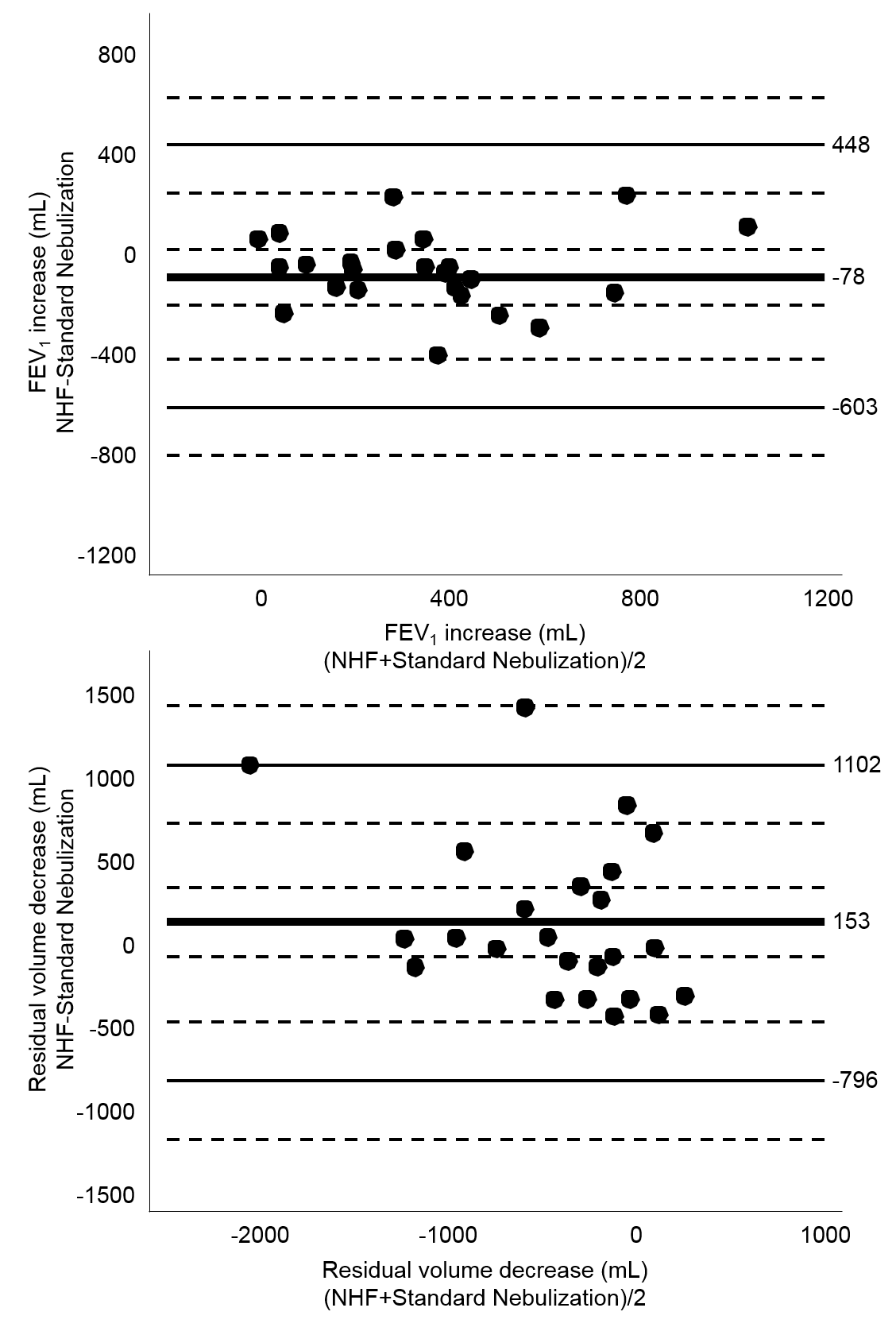


The differences in individual changes in forced expiratory volume in one second (FEV_1_) occurring after nasal high flow-nebulization (NHF-Nebulization) minus the change occurring after Standard-Nebulization are plotted against their mean values (upper panel). Thick continuous line indicates the mean bias between the values, thin continuous lines the 95% limits of concordance, dotted lines represent 95% confidence intervals of those values. Similarly the reduction of residual volume occurring after nebulization is plotted in the lower panel.

Table E2 shows detailed individual variables of the 5 patients who exhibited a significant airflow obstruction reversibility meeting guidelines definition of reversibility (i.e. increase in FEV1 of at least 12% and 200 mL).

| Table E2: Individual data of patients showing airflow obstruction reversibility after Control-NHF | | | | | |
| --- | --- | --- | --- | --- | --- |
| Patient number | 2 | 7 | 14 | 15 | 22 |
| Baseline characteristics |  |  |  |  |  |
| Age (years) | 76 | 49 | 74 | 48 | 48 |
| Gender | Female | Male | Male | Male | Male |
| Medical history | Hypertension, COPD | COPD | Asthma | Peripheral arterial disease, COPD | Asthma |
| Weight (Kg) | 60 | 88 | 78 | 67 | 64 |
| Height (cm) | 152 | 174 | 165 | 178 | 175 |
| Body mass index (Kg/m^2^) | 26.0 | 29.1 | 28.7 | 21.1 | 20.9 |
| Expiratory flow limitation | NO | NO | YES | NO | NO |
| Hemodynamic and respiratory variables before/after NHF | | | | | |
| Systolic-Diastolic arterial pressure (mmHg) | 140-84/128-76 | 133-75/133-77 | 166-82/165-84 | 122-77/138-82 | 134-80/112-77 |
| Heart rate (beats/min) | 84/77 | 62/67 | 62/65 | 67/66 | 81/73 |
| Respiratory rate (cycles/min) | 18/14 | 30/24 | 18/18 | 20/28 | 24/24 |
| Pulmonary function tests before/after NHF |  |  |  |  |  |
| FEV_1_ (L) | 0.94/1.16 | 2.57/3.09 | 1.62/1.93 | 1.95/2.32 | 1.99/2.23 |
| Absolut change in FEV_1_(mL) | 220 | 520 | 310 | 370 | 240 |
| Relative change in FEV_1_ (%) | 23 | 20 | 19 | 19 | 12 |
| Functional residual capacity (L) | 4.58/4.26 | 3.80/3.01 | 7.40/7.11 | 4.71/5.07 | 5.08/5.20 |
| Forced vital capacity (L) | 2.16/2.43 | 4.52/4.61 | 3.26/3.51 | 5.00/5.25 | 4.26/4.39 |
| Residual volume before/after NHF (L) | 3.92/3.44 | 2.70/1.69 | 3.99/4.56 | 5.49/5.51 | 2.94/3.60 |
| Plethysmographic airway resistances (raw) | 4.68/3.33 | 7.02/5.26 | 8.63/7.52 | 5.54/4.64 | 4.62/5.51 |
| Inspiratory capacity (L) | 1.52/1.62 | 3.41/3.37 | 3.09/3.68 | 2.49/2.91 | 2.58/2.87 |
| Part III of the volumetric capnography slope | 1.52/1.91 | 0.83/0.40 | 0.35/0.38 | Missing data | 0.75/0.87 |
| COPD: chronic obstructive pulmonary disease; NHF: nasal high flow; FEV_1_: forced expiratory volume in one second | | | | | |
